# Surgical complications in colorectal cancer patients

**DOI:** 10.1016/j.amsu.2020.04.024

**Published:** 2020-05-11

**Authors:** Haleh Pak, Leila Haji Maghsoudi, Ali Soltanian, Farshid Gholami

**Affiliations:** a Department of Surgery, School of Medicine, Shahid Madani Hospital, Alborz University of Medical Sciences, Karaj, Iran; b Department of Anesthesiology, School of Medicine, Alborz University of Medical Sciences, Karaj, Iran

**Keywords:** Colorectal, Surgery, Laparoscopic, Cancer

## Abstract

**Background:**

Advancements in diagnostic and therapeutic sciences have allowed early diagnosis and treatment of cancer. Colorectal cancer is one of the most commonly reported cancers, particularly in elderly patients.

**Methods:**

Open and laparoscopic surgeries are used for the removal of the tumor, along with chemotherapy, depending on the stage of cancer. However, colorectal cancer surgery is associated with a great number of complications, that affect the efficacy of the surgery and overall health and survival of the patient.

**Results:**

Prevalence of these complications have shown discrepancies depending on the condition of the patient and disease and surgical skills of the surgeon. Preoperative evaluation, intraoperative care and postoperative measures can reduce the incidence of these complications.

**Conclusion:**

This review highlights some frequently reported complications associated with colorectal cancer surgery, their risk factors and subsequent therapeutic measures to treat them.

## Highlights

• Colorectal cancer is one of the most commonly reported cancers, particularly in elderly patients.
• Numerous postoperative complications are reported that can significantly lead to morbidities, prolonged hospital stay and even mortality.
• Management of these adverse effects can be beneficial for overall survival rate and improvement in the quality of life.

## 1 Colorectal cancer (CRC) surgery

**Fig. 1:**
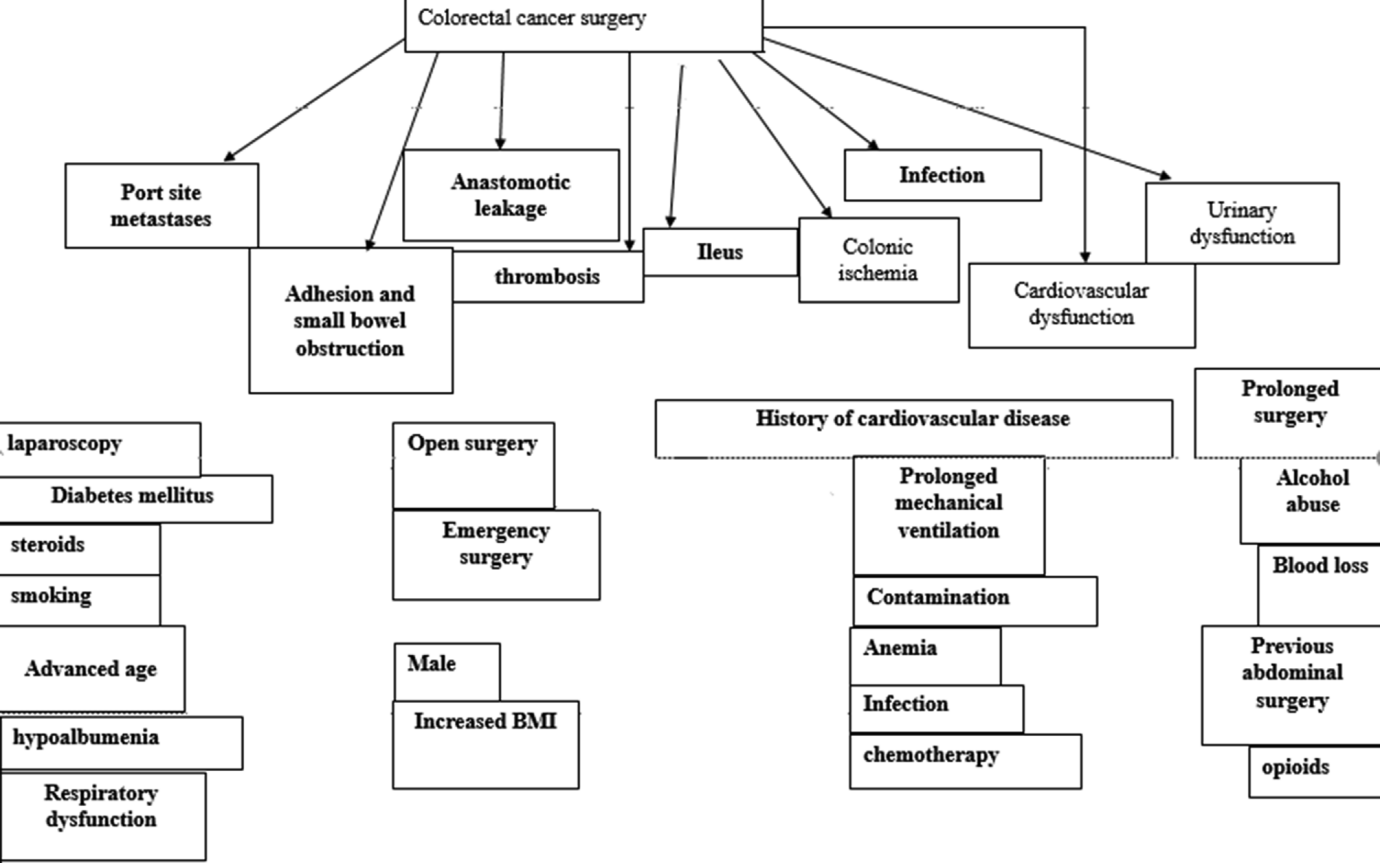
Shows complications associated with colorectal cancer surgery and associated risk factors. Each complication is denoted by a color and corresponding risk factors are shaded.

For the past 50 years, mortality due to colorectal cancer has been increased up to 10 folds. Seventy-five percent of these cases are sporadic however, patients with a familial history of colorectal cancer have a reduced life span. Factors such as; aging, smoking, alcohol abuse, poor diet, increased BMI, diabetes mellitus and diseases like helicobacter pylori infection and Lynch syndrome (familial) significantly contribute to the onset of CRC. Mutations in adenomatous polyposis coli (APC) gene in familial adenomatous polyposis, Adenine DNA mutY gene in Peutz–Jeghers syndrome and chronic inflammatory bowel disease are also identified causes of CRC[1]. The mechanism of colorectal cancer is similar to any other cancer-types. Genetic and epigenetic alterations result in the hyper-proliferation of immunotolerant, apoptosis-resistant and genomically unstable cells cause polyps formation that turn into adenoma and progress into advanced stages. Different types of colonoscopy and other imaging methods are used for the diagnosis CRC [2]. (see Fig. 1)

Treatment strategies vary in accordance with the stage and the type of cancer such as; endoscopy for macroscopic intra-mucosal carcinoma, surgical lymph node dissection, laparoscopic surgery and surgery with chemotherapy metastasized (stage IV and recurrent tumor) [3,4]. The surgery involves removal of the tumor and associated lymph nodes. Two commonly practiced resection methods are open and laparoscopic resection. Similarly, minimally invasive transanal endoscopic microsurgery and total mesorectal excision are performed for the complete removal of the rectal tumor. Whereas, local recurrence of the tumor is avoided by radiotherapy [2].

Heated/hyeprthermic intraperitoneal chemotherapy (HIPEC) is also performed for the removal of tumor (cytoreductive surgery) in peritoneal carcinomatosis, with 16% of curable odds [5]. Owing to the complexities of the tumor-removal surgeries, several complications are notably reported.

## 2 Adhesion and small bowel obstruction (SBO)

Adhesions are the most frequently reported complication associated with laparoscopy, that affect almost 95% of the cases and are the major cause of small bowel obstruction. Other risk factors leading to SBO are; male sex, emergency surgery, longer duration of surgery, open colorectal surgery and dysfunctioning ileostomy placement [6]. Approximately, 10% of colorectal surgery results in SBO along with peritoneal adhesions, postoperatively. A study has shown that laparoscopic and open surgery are equally associated with the development of SBO [7]. Recurrence of adhesive SBO is characterized by a reduced survival rate. Thereby, timely surgical management is required to suppress its recurrence [8].

Respiratory dysfunction test before the surgery can be used as a predictor for postoperative complications including; obstructive bowel and infections [9]. In a recent study, it was revealed that SBO is characterized by re-hospitalization and mortality in 65% of the cases with laparoscopic and open colorectal surgery, within a 5-year period of the study. However other mortality-related factors such as smoking, cardiovascular disease and age were also prevalent [10]. Non-surgical management of SBO is usually performed initially by the means of bowel decompression, however, a considerable number of patients are known to require surgery. Laparoscopic adhesiolysis has superior outcomes in this regard, with lower mortality and fast recovery [11]. Notwithstanding, it is reported to present a higher recurrence rate [12].

Several intraoperative strategies are suggested to minimize the formation of adhesions thereby, preventing the risk of SBO. Seprafilm, bioresorbable films, composed of carboxymethyl cellulose sodium hyaluronate, carboxymethyl cellulose and hyaluronic acid are reported to prevent bowel obstruction and minimize the occurrence of postoperative adverse events [13,14]. Poly(l-lactide-co-D,l-lactide) adhesion barrier is also beneficial against the formation of peristomal adhesion [15]. Nevertheless, variabilities in the outcomes are likely to be the resultant of the surgeon's expertise, skills and period of non-operative patient management [16].

## 3 Thrombosis

The incidence of venous thromboembolic events (VTE) in colorectal surgery patients constitutes to almost 2.5% of the cases. An increased body mass index, anemia, surgical infection, sepsis, lengthy ventilation, irritable bowel syndrome and age are some of the commonly reported risk factors [17,18]. Patients under steroids, history of preoperative sepsis and weight loss, longer surgical duration and postoperative chemotherapy are concomitant with greater risk of venous thromboembolic events [28, 29]. A large cohort study showed that venous thromboembolism is common upon port implantation in these cancer patients, including colorectal cancer [19]. Xie, Fang [20] conducted a meta-analysis of 9 randomized trials based on approximately 2600 CRC patients, to investigate the incidence of deep vein thrombosis in laparoscopic and open colorectal surgery. Both the procedures were associated with the risk of thrombosis, despite not statistically significant, risk in laparoscopic method was lesser.

Preoperative screening of patients undergoing these CRC surgery can reduce the intra and post-operative complications [21].

Guidelines have suggested the use of anti-thrombotic therapy (pharmacological thromboprophylaxis) such as, prophylactic low molecular weight heparin (LMWH) for the prevention of thrombosis [22,23]. Extended thromboprophylaxis, for 30 days, starting from the perioperative period, can reduce thromboembolic events, as compared to 10 days standard LMWH therapy [24].

Graduated compression stockings (GCS) is also studied widely for the prevention of intra and post-operative thrombosis. Despite older studies have reported advantageous outcomes of GSC [25], recent studies have failed to report a reduction in the incidence of VTE with prophylactic compressions [26]. Comparative analysis of pharmacological and mechanical thromboprophylaxis and the use of combinational therapy can demonstrate better outcomes [27].

## 4 Infections

Postoperative infections contribute predominantly to the morbidity and mortality related to colorectal surgery. Surgical site infections (SSI) associated with colorectal surgery are 4 times more than any other abdominal surgery. Four frequently reported factors leading to a higher incidence of infections include; advanced age, perioperative complications leading to morbidity, type of surgical wound (clean, clean-contaminated, contaminated or dirty) and surgeries for neoplasm. Other factors such as diabetes mellitus, chemotherapy and steroid use can also increase the risk of SSI [28]. Hence, several strategies are defined to prevent infection during the surgery [29].

Mechanical bowel preparation (MBP) is recommended for patients undergoing CRC surgery, which is targeted to clear fecal matter from large bowel in order to prevent complications; such as sepsis. This is achieved through bowel clearing agents like enemas, laxatives, cathartic, polyethylene glycol and sodium [30]. Studies have shown that MBP can reduce postoperative complications such as; infection, anastomotic leak and ileus [31]. However, the exemption of MBP may have no effect on the incidence of morbidities [32]. Pharmacological interventions include; IV and oral antibiotics to prevent surgical site infection [33]. Gomila, Carratala [34] in their recent study found that Pseudomonas aeruginosa infection is a common cause of surgical site infection and is associated with poor postoperative results. They suggested that preoperative prophylactic use of antibiotics is likely to improve these outcomes.

MBP with antibiotics, hospital staff training and entry-exit management of operation theater significantly reduce surgical site infection, postoperative complications (Clavien Dindo stage III-V) and duration of hospital stay [29]. It can be presumed that human-based negligence can cause wound infections. Several other such bundles of preoperative and perioperative measures can reduce SSI after colorectal surgery up to 40% [35,36]. A recent meta-analysis of 665 patients presenting SSI after colorectal surgery showed that antibiotic lavage, ionized silver dressing on the closed abdominal wound and topical application of vitamin E oil on subcutaneous tissue are effective strategies to prevent wound infection [37].

Peritonitis and sepsis are also reported after CRC surgery. Peritonitis is usually managed by relaparoscopic surgery, however, is associated with complications [38,39]. Sepsis can lead to deep vein thrombosis [40] and reduced survival rate [41]. Preoperative hypoalbuminemia is associated with an increased prevalence of sepsis [42] whereas, an intake of probiotics and synbiotics can reduce sepsis up to 38% [43].

## 5 Port site metastases

Port site metastases are commonly associated with minimally invasive resection of colorectal surgery [44]. Laparoscopic surgeries are known to contribute to reduced hospitalization and blood loss, however, overall survival rate, morbidity and mortality are same as open surgery. It is also safe and feasible in geriatric patients and those with previous history of abdominal surgeries [45,46]. Yet, frailty in elderly patients can lead to adverse postoperative outcome [47]. Inconsistencies are reported regarding the outcomes of laparoscopic surgery, perhaps due to the level of expertise of the operator, patients' perioperative conditions and genetic and demographic factors. Laparoscopic surgery is characterized by a reduced incidence of port site metastases [48]. Additionally, single-incision laparoscopy reduces pain and blood loss, has shorter incision and hospitalization duration in CRC patients. Nonetheless, it is not a cost-effective alternative to conventional laparoscopy [49]. Hand-assisted laparoscopy for CRC has been reported to have superior outcomes in terms of survival rate, postoperative complications and port site metastases [50].

A recent animal study has shown that peritoneal dissemination can be prevented during laparoscopic surgery by hyperthermic insufflation using CO_2_. It can decrease the incidence of port site metastases and count and weight of peritoneal nodules [51]. Hot humified CO_2_ also results in the reduction of an inflammatory response and mesothelial cell injury [52]. Nonetheless, these techniques can lead to wound infection [53].

## 6 Anastomotic leakage (AL)

Anastomotic leak is also one of the frequently reported complications of colorectal surgery, that can cause mortality and various morbidities. According to Colon Leakage Score, male gender, smoking, increased BMI, overuse of alcohol, NSAIDs and steroids usage, emergency surgery and contamination are some of the contributing risk factors that can lead to AL [54]. Charlson Comorbidity Index (CCI) is also a scoring system used for a similar purpose [55]. The prevalence of AL could be from 1.8 to 19.2%, depending on the pre and intra-operative risk factors such as blood loss, changes in blood pressure and contamination [56]. Depending on the severity of the leakage, it is divided into three grades A, B and C, respectively. Grade A and B can be managed non-surgically via antibiotics and tubal draining, however, grade C usually requires reoperation and might result in 3 or more complications including, mortality [57]. Not only it is associated with decreased overall survival, but can also increase the risk of cancer recurrence [58]. Nagib, Kiffin [59] presented a case where necrotizing fasciitis was diagnosed in a geriatric patient after 8 years of colorectal resection, and late diagnosis resulted in spreading of the infection and death.

Treatment of AL is determined based on the size and location of the leak site, overall condition of the patient, presence of nearby lymph node and cause of primary resection. Endoluminal vacuum‐assisted therapy (EVT) is one of the minimally invasive methods to drain AL, where, polyurethane sponge is endoscopically inserted in the leak site to drain and reduce the size of the defect. Shalaby, Emile [60] reported that EVT is an effective strategy to correct AL with the restoration of bowel rejoining with stoma. Whereas, conventional transanal tube drainage is cheap, safe and effective too, following rectal resection [61]. Laparoscopic low and ultralow anterior resection can also reduce the incidence of AL after rectal cancer surgery [62].

Nickel-titanium ring (NiTi CAR 27) is used for anastomosis instead of sutures or staples to avoid these complications. Studies have shown that these rings are equally efficient as compared to traditional suturing techniques [63,64]. Other under-consideration techniques to strengthen anastomosis include; gelatin sealant, cyanoacrylate adhesives, omental wrapping and mesenteric flaps [54]. Assessing the success of anastomosis during surgery needs meticulous consideration thereby, several techniques are practiced by the surgeons for the purpose so prevent postoperative AL. CT scan is conventionally exploited to detect the leak nonetheless, it could direct false negative results which can delay the therapeutic intervention and cause further complications [65]. Fluorescence angiography using indocyanine green can reduce AL in colorectal surgeries [66]. Similarly, measurement of perioperative colonic oxygen saturation ≤90%, using pulse oximeter, is a significant indicator for detecting leakage [67]. Biomarkers such as; c-reactive protein, white blood cell count, procalcitonin levels are marked with significant postoperative variations after anastomotic leak [68].

## 7 Ileus

Dysfunctioning of intestinal peristalsis as a result of abdominal surgery and anesthesia is known as ileus. It is a common consequence of colorectal surgery and can cause nausea/vomiting, pain and failure of oral food intake. Its incidence varies according to the type and length of the procedure [69], preoperative conditions and the emergency of the operation. Risk factors of ileus include; advanced age, increased BMI, smoking, alcohol abuse, history of previous abdominal operation, use of opioids, blood loss, peripheral vascular disease, respiratory dysfunction and adhesions from previous surgeries [[70], [71], [72]]. Furthermore, prolonged postoperative ileus can lead to anastomotic leakage and intra-abdominal infections [73].

Yang, Zuo [74] showed that acupuncture and simo decoction (Chinese medicine) can reduce postoperative ileus in colorectal cancer resection patients and decrease the duration of the hospital stay. Similarly, MBP is effective to reduce the prevalence of ileus [75]. Robot-assisted colectomy can also lessen ileus and other above-mentioned complications following CRC surgery [76].

Laparoscopic surgery enhances Treg response and is associated with a lower incidence of ileus as compared to open colorectal surgery [77]. Inflammation due to surgery can affect sympathetic feedback from the nervous system, which forms the underlying mechanism of ileus. These patients have increased levels of TNF-α (tumor necrosis factor-alpha) and c-reactive protein, 2 days after the surgery [78]. A meta-analysis concluded that stimulation of the autonomic nervous system by chewing gum is a cheap and effective way to prevent ileus [79]. Elevated inflammatory response in postoperative ileus subjects is also associated with greater risk of the anastomotic leak [78].

## 8 Colonic ischemia (CI)

Colonic ischemia, also known as ischemic colitis, is an unusual yet serious complication after the colorectal surgery. Old age, male gender and preexisting cardiovascular pathologies constitute the greater incidence of CI in patients undergoing colorectal cancer surgery [80]. It can also occur in patients undergoing reoperation via laparoscopy for peritonitis [38,81]. Ikeda, Takahashi [82] recently reported a case of an elderly women presenting infrarenal aortic stenosis with colorectal cancer. She underwent colon resection, 2-year after which she developed colonic ischemia as a result of thrombus formation in marginal artery. Laparoscopy was performed to remove the obstruction due to thrombosis. It is evident that previous cardiovascular pathology can worsen the complications after colectomy and can lead to ischemic colitis.

## 9 Other system complications

A great population of individuals undergoing colorectal surgery comprises of geriatric patients. Advanced age is associated with systemic aging and intolerance to trauma. Therefore, complications of colorectal cancer surgery are chiefly seen in elderly patients such as respiratory and cardiovascular adverse events. These complications are associated with an increased risk of 30-day postoperative mortality [83]. Hypoalbuminemia, that is a common preoperative condition in CRC patients, can to adverse respiratory events such as; requirement of mechanical ventilation and intubation [84].

Laparoscopic surgery can reduce the incidence of these adverse events [85,86]. To it, preoperative exercise in patients undergoing surgery can also reduce adverse cardiopulmonary events [87]. Usage of beta-blockers before the surgery is found effective in reducing cardiac complication and corresponding postoperative mortality [88].

Depending on the metastases of cancer, often during surgical resection, parts of the urinary tract are resected, thereby disrupting the system[89, 90]. Bolmstrand, Nilsson [91] in their recent study reported that patients who underwent urinary bladder and ureter resection in colorectal cancer surgery were marked with 22% of urological complications such as; wound dehiscence and urinary leak, resulting in the treatment with permanent nephrostomy tubes. Iatrogenic ureteral injuries are also reported as a result of laparoscopic surgery [92]. Age, laparoscopic surgery and abdominoperineal resection are risk factors associated with postoperative urinary retention. 4% of colorectal cancer surgery patients are at the risk of developing urinary tract infection particularly, geriatric females undergoing the rectal procedure with preoperative steroid use, prolonged duration of the surgery and under higher classes of anesthesia. These patients have a longer duration of hospitalization and greater incidence of postoperative 30-day mortality [93,94]. However, urinary dysfunction can also occur without the prevalence of any risk factor [95] Invasion of cancer with urinary tract accompanies negative resection margin challenge. This can lead to the recurrence of the tumor, increased morbidity and the spread of cancer [96]. In order to avoid these complications, complete bladder removal is required, that can also compromise patients' quality of life [97]. Frail elderly patients with the age of 75 and above are more prone to acquire urinary tract-related postoperative complications [98].

## 10 Conclusion

Past few years have been marked with an increased incidence of colorectal cancer. Surgical management has been successfully exploited to remove and treat the cancer however, numerous postoperative complications are reported that can significantly lead to morbidities, prolonged hospitalization and mortality. Pre and intra-operative risk factors can predict the incidence of these complications. Preexisting comorbidities, advanced age, increased BMI, stage and metastases of cancer and neoadjuvant therapy are the common factors that should evaluated by clinicians and surgeons.

Additionally, management of these adverse effects can be beneficial for overall survival rate and improved quality of life.

## Provenance and peer review

Not commissioned, externally peer reviewed.

## Ethical approval

Research studies involving patients require ethical approval. Please state whether approval has been given, name the relevant ethics committee and the state the reference number for their judgement.

All procedures performed in this study involving human participants were in accordance with the ethical standards of the institutional and/or national research committee and with the 1964 Helsinki Declaration and its later amendments or comparable ethical standards.

## Author contribution

Dr. Haleh Pak: conceptualized and designed the study, drafted the initial manuscript, and reviewed and revised the manuscript.

Dr. Leila Haji Maghsoudi:Designed the data collection instruments, collected data, carried out the initial analyses, and reviewed and revised the manuscript.

Dr. Ali Soltanian and Dr.Farshid Gholami: Coordinated and supervised data collection, and critically reviewed the manuscript for important intellectual content.

## Human and animal rights

No animals were used in this research. All human research procedures followed were in accordance with the ethical standards of the committee responsible for human experimentation (institutional and national), and with the Helsinki Declaration of 1975, as revised in 2013. This study was approved by the Research Ethics Board of Alborz University of Medical Sciences.

## Consent for publication

Informed consent was obtained from each participant.

## Availability of data and materials

All relevant data and materials are provided with in manuscript.

## Funding

None.

## Guarantor

The Guarantor is the one or more people who accept full responsibility for the work and/or the conduct of the study, had access to the data, and controlled the decision to publish.

## Declaration of competing interest

The authors deny any conflict of interest in any terms or by any means during the study. All the fees provided by research center fund and deployed accordingly.
